# Migrants from Low-Income Countries have Higher Heat-Health Risk Profiles Compared to Native Workers in Agriculture

**DOI:** 10.1007/s10903-023-01493-2

**Published:** 2023-05-20

**Authors:** Leonidas G. Ioannou, Davide J. Testa, Lydia Tsoutsoubi, Konstantinos Mantzios, Giorgos Gkikas, Gerasimos Agaliotis, Lars Nybo, Zahra Babar, Andreas D. Flouris

**Affiliations:** 1 FAME Laboratory, Department of Physical Education and Sport Science, University of Thessaly, Karies, 42100 Trikala, Greece; 2 Department of Nutrition, Exercise and Sports, August Krogh Building, University of Copenhagen, Copenhagen, Denmark; 3 Center for International and Regional Studies, Georgetown University, Doha, Qatar

**Keywords:** Migrants, Workers, Heat stress, Heat strain

## Abstract

The present observational study was conducted to uncover potential differences in the risk of experiencing high occupational heat strain during agriculture work between migrants and their native coworkers, as well as to elucidate the factors that may contribute to such differences. The study took place over the period from 2016 through 2019 and involved monitoring 124 experienced and acclimatized individuals from high-income (HICs), upper-middle-income (UMICs), as well as lower-middle- and low-income (LMICs) countries. Baseline self-reported data for age, body stature, and body mass were collected at the start of the study. Second-by-second video recordings throughout the work shifts were captured using a video camera and were used to estimate workers’ clothing insulation, covered body surface area, and body posture, as well as to calculate their walking speed, the amount of time they spent on different activities (and their intensity) and unplanned breaks throughout their work shifts. All information derived from the video data was used to calculate the physiological heat strain experienced by the workers. The core temperature of migrant workers from LMICs (37.81 ± 0.38 °C) and UMICs (37.71 ± 0.35 °C) was estimated to be significantly higher compared to the core temperature of native workers from HICs (37.60 ± 0.29 °C) (p < 0.001). Moreover, migrant workers from LMICs faced a 52% and 80% higher risk for experiencing core body temperature above the safety threshold of 38 °C compared to migrant workers from UMICs and native workers from HICs, respectively. Our findings show that migrant workers originating from LMICs experience higher levels of occupational heat strain, as compared to migrant workers from UMICs and native workers from HICs, because they take fewer unplanned breaks during work, they work at a higher intensity, they wear more clothing, and they have a smaller body size.

## Background

Global warming is accompanied by adverse climatic conditions such as prolonged drought and heat stress periods [1–3] with marked effects on agriculture and potentially disrupting major food supply chains [4]. The economic implications related to these events [5, 6] and the consequences on food security [7, 8] are becoming increasingly apparent. However, what is often neglected is the impact of global warming on the health and well-being of those involved in the cultivation of crops as well as in other jobs throughout the agriculture industry. Billions of workers are currently exposed to increased occupational heat stress on a daily basis [9, 10], and these estimates will increase in the coming years due to global warming [9].

Communication between experts and policy-makers [11] and adoption of heat mitigation strategies [12–14] are currently taking place to address the effects of heat exposure on the health and well-being of agriculture workers. In the last decades, the employment of migrant workers in agriculture has drastically increased in some European countries [15]. This created the need to investigate if their new work environment impacts their health and well-being, considering potential differences from the local population. Inability to speak the local language as well as lack or limited technical skills and access to occupational health and safety training create significant barriers in their everyday work activities and increase their susceptibility to injury and illness [16–19]. Therefore, it is not surprising that migrant workers experience heat-related illnesses more frequently [18, 20–22], and that they report performing jobs requiring more effort compared to native workers [23]. Although these findings are undoubtedly a step forward in migration research, they are exclusively based on self-reported information obtained through questionnaires and surveys and may not uncover the full picture. Therefore, the present observational study was conducted to uncover potential differences in the risk of experiencing high occupational heat strain during agriculture work between migrants and their native coworkers, as well as to elucidate the factors that may contribute to such differences.

## Methods

The study involved monitoring 124 experienced (i.e., agriculture is their main source of income) and acclimatized (i.e., continuously living in the area and performing agriculture jobs on a daily basis for ≥ 2 months) agriculture workers. The workers performed a range of activities including grape-picking, carrying, loading, driving trucks/tractors, handling machinery, supervision, etc. It took place over the period from 2016 through 2019 and involved analyzing full work shifts in 17 farms spread across Cyprus. The experimental protocol for the field experiments was approved by the Bioethical Committee of School of Exercise Science of the University of Thessaly (no. 1217) and the National Bioethical Committee of Cyprus (NBCC 27.01.61) in accordance with the Declaration of Helsinki.

Participants were divided into three groups based on the economic development of their country of birth: (1) high-income countries (HICs), (2) upper-middle-income countries (UMICs), as well as (3) lower-middle- and low-income countries (LMICs). This categorization has been used in previous migration studies [24] and was done according to the classification of economies as published in the World Economic Situation and Prospects of the United Nations [25]. To allow comparisons between HICs, UMICs, and LMICs, the study involved monitoring farms that employed workers from two or more of these groups. Workers from UMICs were covered by the Schengen agreement for passport-free travel across Europe, while workers from LMICs were on a 6-year agricultural work permit. Prior to their participation in the study, written informed consent was obtained from all volunteers after detailed explanation of all the procedures involved provided in a language that they could understand.

Baseline self-reported data for age, body stature, and body mass were collected at the start of the study. Body stature and mass were used to calculate the body surface area of the workers using Dubois’ formula [26]. Continuous environmental data [air temperature (°C), globe temperature (°C), relative humidity (%), and air velocity (m/s)] and Wet-Bulb Globe Temperature (WBGT) were collected using a portable weather station (Kestrel 5400FW, Nielsen-Kellerman, Pennsylvania, USA). The WBGT was used because it is the recommended thermal stress indicator for quantifying the heat stress experienced by outdoor workers [27–29].

Based on previously-established methodology [12–14, 30–32], video recordings throughout the work shifts were captured using a video camera (Hero 5 black, GoPro, California, USA) installed in close proximity (~ 40 m) to the workers. To mitigate the impacts of the so-called “Hawthorne effect”, which indicates that workers change aspects of their behaviour as a result of their awareness of being studied [33], we followed the steps described previously [12, 30]. Thereafter, these video footages were used to calculate workers’ clothing insulation (measured in CLO units), covered body surface area (in m^2^), body posture (in m), and walking speed (in m/sec) throughout their work shifts [32]. The same video recordings were analyzed on a second-by-second basis [12, 30], to estimate the amount of time spent on unplanned breaks as well as the work intensity (in W/m^2^) of each worker throughout the entire work shift [12, 30]. Specifically, time-motion analysis was conducted for 11 different activities: unplanned break taking place in the shade (76 W/m^2^), planned lunch break (87 W/m^2^), unplanned break taking place under the sun (116 W/m^2^), giving instructions and guidance to the workers (134 W/m^2^), moving the truck to a different place (146 W/m^2^), driving and/or pushing the mechanical fruit cart (204 W/m^2^), and picking/harvesting crops (204 W/m^2^), helping other workers with their tasks (204 W/m^2^), lifting boxes full of fruits (262 W/m^2^), carrying three to four empty fruit boxes (279 W/m^2^), carrying 25-kg-boxes full of fruits (436 W/m^2^) [34, 35]. As previously, unplanned breaks were expressed as the percent of work shift spent doing non-work related tasks (e.g., resting in shade, drinking water, smoking, etc.) [12, 30, 36]. It is important to note that, after the data collection was completed, all volunteers were notified about the actual purpose of the video recordings and provided their consent for the analysis and publication of the data.

All information derived from the video data was incorporated into the FAME Lab Predicted Heat Strain software, which allows to predict the core body temperature (T_C_), the skin temperature, and the total water loss on a minute-by-minute basis [32]. Thereafter, T_C_ values were used to calculate the percentage of work time spent having T_C_ higher than 38 °C (T_C_^38^), for each worker. The T_C_^38^ safety threshold was adopted following a technical report from the World Health Organization [37] stating that “*it is considered inadvisable for the deep body temperature to exceed 38 °C for prolonged daily exposures in heavy work*”.

To address the main aim of the study, one-way ANOVA with a Bonferroni post hoc adjustment was used to examine possible differences in body surface area, work intensity, unplanned breaks, and clothing insulation among workers originating from HICs, UMICs, and LMICs. To confirm that potential findings were not influenced by potentially extreme situations occurring in some farms (i.e., migrants being highly exploited in certain cases, influencing the trend), the T_C_ data were visually inspected to detect potential differences in the trends across the 17 farms. Pearson’s correlation coefficient was used to detect whether the T_C_, work intensity, or unplanned breaks were associated with the Gross Domestic Product per capita for the country of birth of each worker (obtained from the World Bank on October 7th, 2021) [38]. Risk ratios were utilized to examine potential differences in the risk for experiencing T_C_^38^ among people originating from HICs, UMICs, and LMICs. Statistical analyses were conducted using the SPSS v28.0 (IBM, Armonk, NY, USA) and Excel spreadsheets (Microsoft Office, Microsoft, Washington, USA). The level of significance for all analyses was set at p < 0.05. All values are presented as (mean ± SD), unless otherwise stated.

## Results

Thirty-two native European workers from Cyprus were grouped as being from HICs (sex: 26 males, 6 females; age: 51.7 ± 16.7 years; body stature: 172.8 ± 9.4 cm; body mass: 86.5 ± 15.8 kg) and 43 workers from the Balkan peninsula (Bulgaria: 3; Romania: 40) were grouped as being originated from UMICs (sex: 25 males, 18 females; age: 45.2 ± 8.1 years; body stature: 167.5 ± 6.4 cm; body mass: 73.4 ± 17.9 kg). Forty-nine workers from four south-east Asian countries (Bangladesh: 1; India: 18; Philippines: 12; and Vietnam 18) were grouped as being from LMICs (sex: 24 males, 25 females; age: 34.1 ± 8.2 years; body stature: 163.6 ± 11.9 cm; body mass: 65.1 ± 17.6 kg).

The monitored workers performed their duties in environments ranging from cool to hot (range: 14.6 to 32.5 °C WBGT; average 24.8 ± 4.2 °C WBGT). Ambient conditions throughout the experiments were as follows: air temperature (26.0 ± 4.3 °C; range: 15.9 to 33.3 °C), relative humidity (58.5 ± 17.9%; range: 23.0 to 100%), solar radiation (35.8 ± 8.8 °C globe temperature; range: 16.6 to 50.5 °C globe temperature), and wind speed (1.6 ± 1.6 m/s; range: 0.0 to 7.9 m/s).

**Fig. 1:**
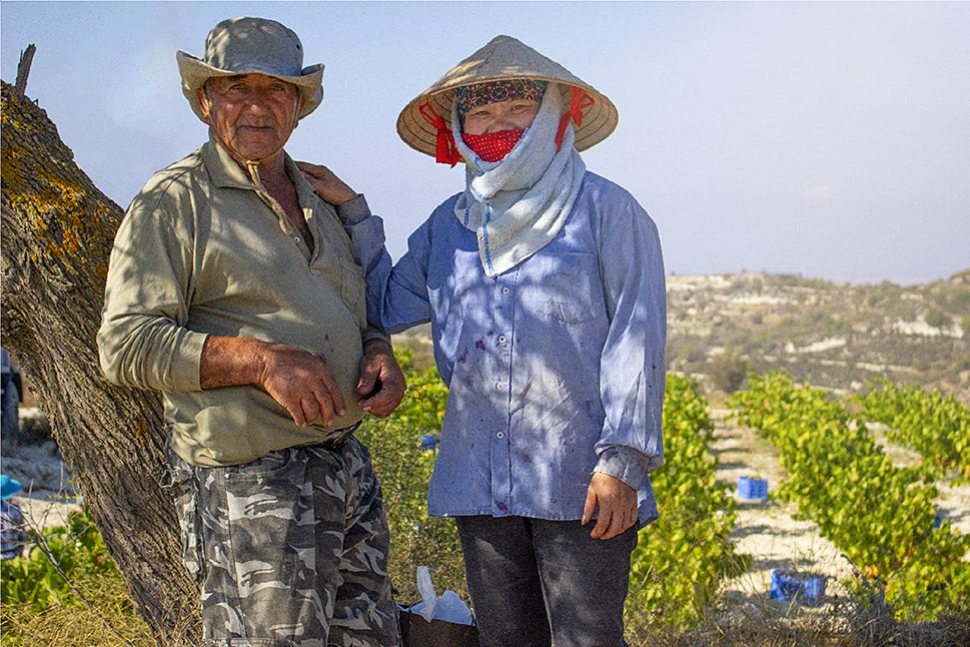
Differences in clothing insulation between a worker from a low-income country (right) and a worker from a high-income country (left). It is important to note that the worker from the low-income country wore multiple layers of clothing covering her head and torso, and that the photo was taken before the COVID-19 pandemic, therefore no facemask was required. Photograph used with permission from the two workers featured

Our analyses detected four main findings. Firstly, that migrant workers from LMICs spent less time on unplanned breaks (9.5 ± 0.8% of work shift), compared to both migrant workers from UMICs (11.5 ± 0.7% of work shift) and native workers from HICs (12.8 ± 1.1% of work shift). The second main finding was that migrant workers from LMICs (198.0 ± 17.8 W/m^2^) and UMICs (195.6 ± 18.1 W/m^2^) performed their work at higher average intensity compared to native workers from HICs (182.0 ± 21.1 W/m^2^) (Table 1; p < 0.05). This is because migrants from LMICs and UMICs were engaged in more physically demanding tasks, such as lifting and carrying heavy boxes of fruits, compared to workers from HICs who performed less physically intense tasks, such as providing instructions and guidance to workers and moving trucks to different locations. The third main finding was that migrant workers originating from LMICs wore significantly more clothing (0.75 ± 0.11 CLO) than migrant workers from UMICs (0.70 ± 0.13 CLO) and native workers from HICs (0.66 ± 0.16 CLO) (Table 2; Fig. 1; p < 0.05). The fourth main finding was that the studied migrant workers [i.e., people from LMICs (1.70 ± 0.28 m^2^) and UMICs (1.82 ± 0.21 m^2^)] had significantly smaller body surface area compared to native workers from HICs (1.97 ± 0.24 m^2^) (Table 3; p < 0.05).

**Table 1:**
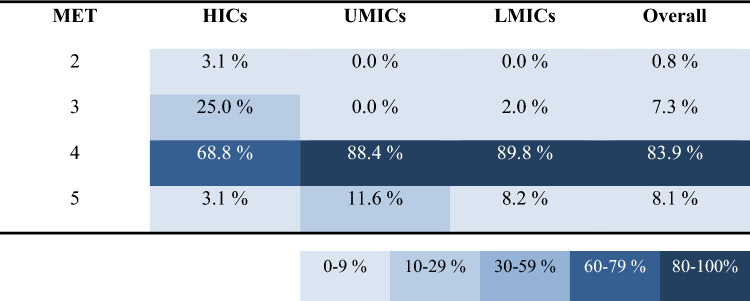
Distribution of work intensity between people across workers originating from high-income (HICs), upper-middle-income (UMICs), as well as lower-middle- and low-income (LMICs) countries

Metabolic equivalent (MET) = 1 corresponds to resting condition, MET = 2 represents work twice as demanding as being in resting condition, and so forth

**Table 2:**
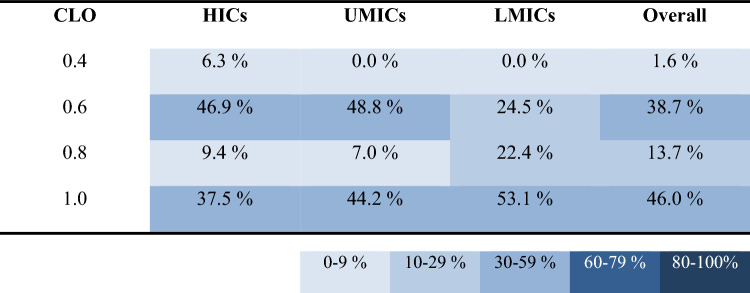
Distribution of clothing insulation (expressed in CLO units) across workers originating from high-income (HICs), upper-middle-income (UMICs), as well as lower-middle- and low-income (LMICs) countries

**Table 3:**
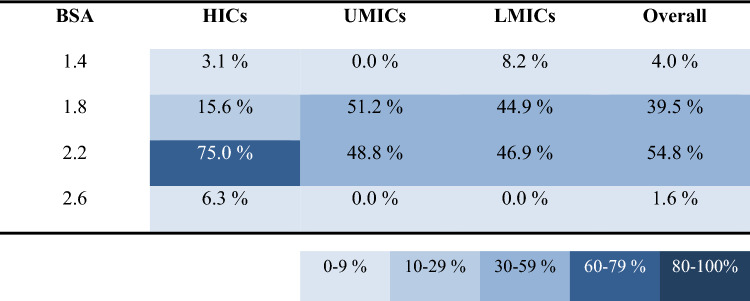
Distribution of body surface area (BSA; expressed in m^2^) categories, across workers originating from high-income (HICs), upper-middle-income (UMICs), as well as lower-middle- and low-income (LMICs) countries

**Fig. 2:**
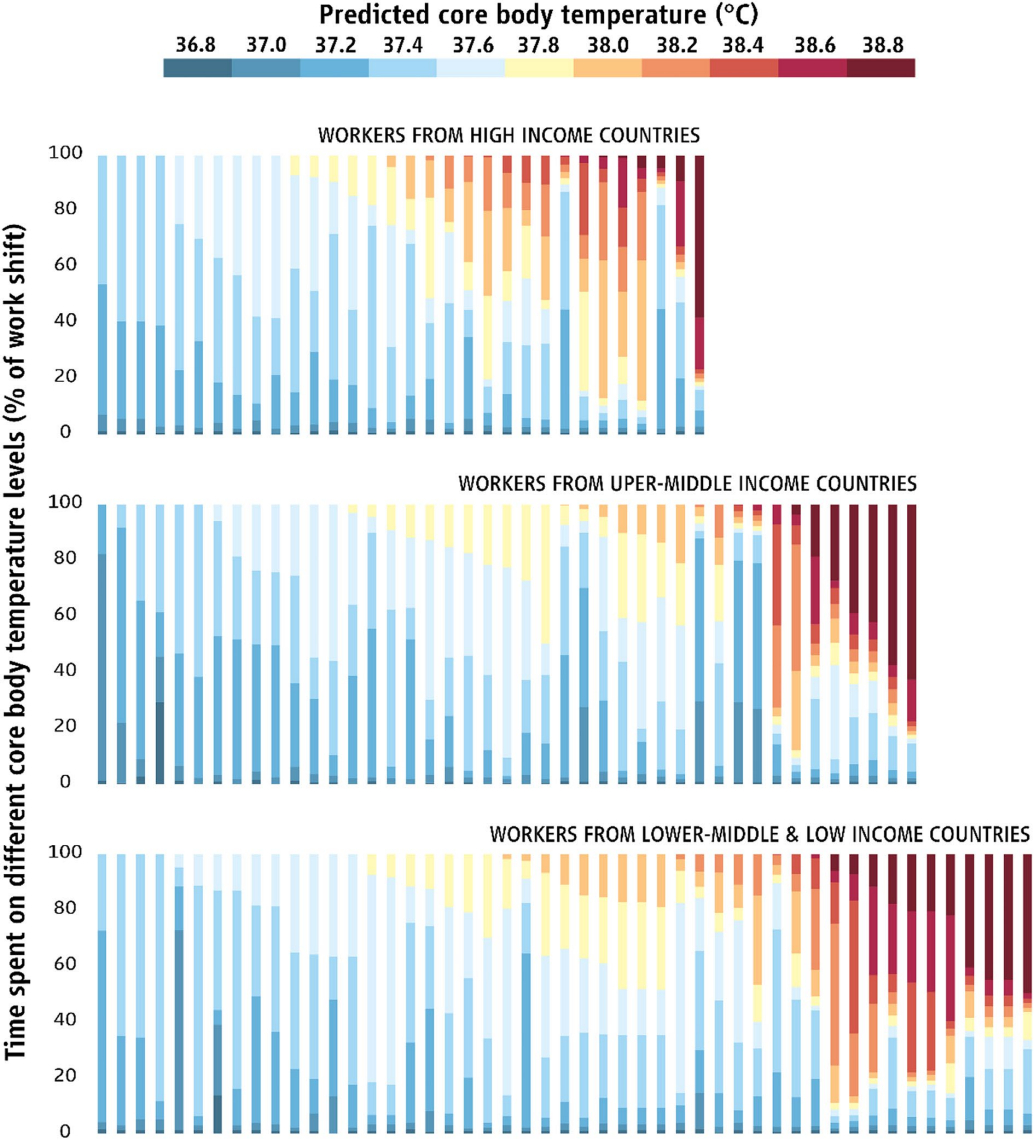
Time spent at different levels of predicted core body temperature during the work shift for workers originating from high-income, upper-middle-income, as well as lower-middle- and low-income countries. Each bar represents a different worker

The four main findings presented above had drastic effects on the physiological heat strain experienced by workers across the three groups, whereby migrant workers from LMICs and UMICs experienced greater heat strain compared to native workers from HICs (Fig. 2). Specifically, the T_C_ of migrant workers from LMICs (37.81 ± 0.38 °C) and UMICs (37.71 ± 0.35 °C) was significantly higher compared to the T_C_ of native workers from HICs (37.60 ± 0.29 °C) [F_(2, 87)_ = 11.84, p < 0.001]. Moreover, migrant workers from LMICs faced a 52% [95% CI (3% to 125%)] and 80% [95% CI (10% to 193%)] higher risk for experiencing T_C_^38^ compared to migrant workers from UMICs and native workers from HICs, respectively. Similarly, on average, workers from LMICs spent significantly more time beyond the T_C_^38^ (27.7 ± 31.3% of work shift) compared to migrant workers from UMICs (17.0 ± 27.5% of work shift) and native workers from HICs (13.4 ± 25.4% of work shift) (Fig. 2). These averages reflect the pattern identified across all 17 farms, confirming that our findings of increased occupational heat strain among migrant workers from low-income countries do not represent sporadic cases, but are likely to reflect the conditions experienced by millions of workers.

We found a weak but statistically significant association between Gross Domestic Product per capita for the country of birth of each worker and the amount of time spent on unplanned breaks (r = 0.232, p = 0.01), indicating that workers originating from poorer countries spent less time resting during work. Also, our analyses revealed a modest negative association between Gross Domestic Product per capita for the country of birth of each worker and his/her work intensity (r = − 0.327, p < 0.001), indicating that workers from poor countries worked harder. Finally, there was a weak but statistically significant negative association between the Gross Domestic Product per capita for the country of birth of each worker and the amount of time spent beyond T_C_^38^ (r = − 0.203, p = 0.024), suggesting that workers from poorer countries experienced greater levels of physiological heat strain.

## Discussion

In this study, we report that migrant workers originating from LMICs experience higher levels of occupational heat strain, as compared to migrant workers from UMICs and native workers from HICs. This is driven by four main factors: (1) fewer unplanned breaks taken during work, (2) higher work intensity, (3) more clothing worn, and (4) a smaller body size.

By studying work behaviour on a minute-by-minute basis, our findings show that the studied migrants from LMICs and UMICs worked on average 8% more intensely compared to native workers from HICs. Translating this into a work-related example, this means a migrant worker carrying a 30-kg box of fruits compared to a native worker carrying a 24-kg box (based on the energy cost of each activity) [34]. Assuming that the two workers must carry 100 boxes of fruits during their shift, at the end of the day the studied migrant worker carries 600 kg more compared to his/her native co-worker from HICs.

Self-pacing is the basis of heat mitigation [12, 14]. Our findings demonstrated that, on average, workers from HICs took 61 min of unplanned breaks over an 8-h shift; in contrast to workers from LMICs and UMICs who rested for 45 and 55 min, respectively. In this sense, our findings confirm a previous questionnaire-based study in which migrant workers in Europe stated that their work requires more effort compared to native workers and that they sustain high levels of work output despite the heat [23]. Overall, our findings demonstrate that migrant workers risk their health and well-being by working harder even in conditions of elevated heat stress.

In addition to the above risk factors, the studied migrant workers from LMICs were found to wear clothing with higher insulation compared to their co-workers, a factor that can reduce the heat loss from their body to the surrounding environment [12, 39, 40]. This increased clothing insulation among migrant workers was, to some extent, due to cultural coverings (see Fig. 1) which may enhance the physiological heat strain experienced by agriculture workers. To tackle this important societal problem, we suggest appropriate training and education on the impacts of occupational heat stress on the health and well-being of migrants involved in occupations with increased heat exposure [17, 18]. For instance, wearing light-colored clothing is known to reduce the physiological heat strain experienced by agriculture workers [13], without the need of violating their religious values.

The low body surface area found among migrant workers is known to play an important role in dissipating the excess heat from the body [31, 41], in a way that larger bodies dissipate more heat to the surrounding environment. Changes in body surface area are largely driven by genetics as well as socioenvironmental parameters, including diet quality and the prevalence of infectious diseases, particularly in the early childhood [42–44]. Our findings point towards equity over equality in the allocation of work tasks, in a way that workers are assigned with tasks of similar relative difficulty. For instance, based on the energy cost of each activity [45], a worker with a small body size (height: 165 cm; weight: 70 kg) carrying a 15-kg box of fruits experiences the difficulty that a worker with a large body size (height: 185 cm; weight: 90 kg) experiences when carrying two such boxes, and vice versa.

It is important to note that the present study involved monitoring migrants working in a HIC country in the south-east Mediterranean (i.e., Cyprus), and thus our findings may not reflect the conditions under which migrant workers perform their jobs in other parts of the world. Additionally, our reliance on self-reported anthropometric data may have potential implications for our findings, particularly in relation to the calculation of workers' body surface area and its impact on the exchange of heat between their bodies and the external environment. Furthermore, although our observations involved a comparatively large sample size incorporating workers from many nationalities, more work is necessary to elucidate the health and well-being of migrant workers in the face of climate change. In conclusion, our findings show that migrant workers originating from LMICs experience higher levels of occupational heat strain, as compared to migrant workers from UMICs and native workers from HICs, because they take fewer unplanned breaks during work, they work at a higher intensity, they wear more clothing, and they have a smaller body size. Based on the findings of the present study, policy makers should establish clear and comprehensive regulations that explicitly address the health and safety of migrant workers who are frequently exposed to heat stress at work. These regulations should be comprehensive, considering the unique needs and vulnerabilities of migrant workers to climate hazards, including those identified in the present study as well as potential ones that may arise in the future. A recent example of such regulation related to occupational heat stress was enacted by the Qatar's Ministry of Administrative Development, Labor and Social Affairs requiring employers to provide annual health checks, training and education. The same legislation requires employers to provide extensive cessation of mid-day work, access to shaded areas, and cool drinking water, while it galvanized the rights for workers to take breaks and to be represented in committees that plan the pace and mode of work [14, 46].
